# A PCR-RFLP Assay targeting RPS8 gene for the discrimination between bovine *Babesia* and *Theileria* species in China

**DOI:** 10.1186/s13071-015-1085-x

**Published:** 2015-09-17

**Authors:** Zhancheng Tian, Junzheng Du, Jifei Yang, Aihong Liu, Xiaocui Liu, Guangyuan Liu, Hong Yin

**Affiliations:** State Key Laboratory of Veterinary Etiological Biology, Key Laboratory of Veterinary Parasitology of Gansu Province, Lanzhou Veterinary Research Institute, Chinese Academy of Agricultural Sciences, Lanzhou, 730046 P. R. China; Jiangsu Co-innovation Center for Prevention and Control of Important Animal Infectious Diseases and Zoonoses, Yangzhou, 225009 P. R. China

**Keywords:** Bovine Babesia and Theileria species, Ribosomal protein S8, Discrimination, PCR-RFLP

## Abstract

**Background:**

Bovine babesiosis and theileriosis is an important hemoprotozoal disease in cattles and yaks in tropical and subtropical regions leading to significant economic losses. In the field, the risk of co-infection between the bovine *Babesia* and *Theileria* species is very high. Thus, it is necessary to develop a simple, accurate, rapid and cost-effective method for large-scale epidemic investigation, in particular for the detection of co-infection in field.

**Methods:**

In this study, DNA sequences of a ribosomal protein S8 (RPS8) gene from eight species of cattle piroplasms in China were used to develop a species-specific PCR-RFLP diagnostic tool. The eight *Theileria* and *Babesia* species could be differentiated by digesting the RPS8 PCR product with M*bo* I.

**Results:**

The sensitivity of the PCR assays was 0.1 pg DNA for *Babesia* species but 1 pg DNA for *Theileria* species. The clearly different size of the PCR-RFLP products allowed for a direct discrimination between eight bovine *Theileria* and *Babesia* species (*T. annulata*, *T. sinensis*, *T. sergenti*, *B. ovata*, *B. bovis*, *B. bigemina*, *B. major* and *Babesia* species Kashi isolate).

**Conclusion:**

Our results indicated that the established method based on the RPS8 gene was a reliable molecular diagnostic tool for the simultaneous detection and identification of bovine *Babesia* and *Theileria* species in China, which could be applicable for the survey of parasite dynamics, epidemiological studies as well as prevention and control of the disease.

## Background

Piroplasms, comprising mainly the genera *Babesia* and *Theileria*, are tick-transmitted protozoa that are pathogenic to ruminants, horses, pigs, dogs, cats and cattle, and in some cases, even to humans. In the vertebrate hosts, the infection usually causes fever, anemia and haemoglubinuria, and in severe cases, death [1, 2]. Animals that recover from acute or primary infections remain chronically infected, and act as reservoirs for ticks [3].

Eight species of bovine *Babesia* and *Theileria* (*B. bigemina*, *B. bovis*, *B. major*, *B. ovata*, an unidentified *Babesia* sp., *T. annulata*, *T. sergenti* and *T. sinensis*) have been identified in China [4]. The eight species of bovine *Babesia* and *Theileria* species can cause a significant loss in meat and milk production from cattles in some parts of China. *Boophilus microplus* has been demonstrated to be the vector of *B. bigemina* and *B. bovis* [5], while *Haemaphysalis longicornis* and *H. punctata* are potential vectors of *B. major* [6, 7]. *H. longicornis* is also considered to be the vector of *B. ovata* and *T. sergenti* [4, 8]. Nymphal progeny derived from female *Hyalomma anatolicum anatolicum* collected from the field were shown to be capable of transmitting an unidentified *Babesia* sp. (Designated *Babesia* Usp.) to calves [9]. *Hyalomma* spp., including *Hyalomma detritum*, *Hy. a. anatolicum* and *Hy. rufipes*, are distributed mainly in semi-dry and desert-land in Northern China, and have been reported to be vectors of *T. annulata* [10]. *T. sinensis* is transmitted by *H. qinghaiensis* [11]. In the field, the risk of co-infection between the eight bovine *Babesia* and *Theileria* species is very high. The species are morphylogically indistinguishable, and molecular techniques have become the key to species identification. So it is necessary to develop a simple, reliable and cost-effective method that is suitable for large-scale epidemic investigation, particularly for the detection of co-infection in field [12].

In the work described here, an informative molecular target has been identified in the ribosomal protein S8 (RPS8) gene from bovine *Babesia* and *Theileria* species endemic in China. The amplified gene fragment containing non-coding regions varied extensively both in length and in sequence, and allowed the development of an assay for species differentiation based solely on fragment size when combined with a simple PCR-restriction fragment length polymorphism (RFLP) protocol.

## Methods

### Ethics statement

All animal experiments were performed according to the protocols approved by the Animal Care and Use Committee of the Lanzhou Veterinary Research Institute (permit number 2009–26).

### Parasite species

The isolates used in this study were listed in Table 1. *Babesia bovis* (Shanxian and Lushi) [13], *B. bigemina* (Kunming and Lushi) [14], *B. major* (Yili) [7], *B. ovata* (Wenchuan and Lushi and Zhangjiachuan) [15], *Babesia* sp. Kashi2 (Kashi) [16],*Theileria annulata* (Xingjiang and Ningxia and Sanmenxia) [17], *T. sergenti* (Lushi) [4], *T. sinensis* (Weiyuan and Lintan and Lintao) [18].

**Table 1:** The location, vector and RPS8 (coding and non-coding regions) gene accession numbers for *Babesia* and *Theileria* species used in this study

Parasite	Location	Tick vector	RPS8 Accession No.
*Babesia bovis*	Shanxian	*Boophilus microplus*	JN400408
*B. bovis*	Lushi	*B. microplus*	JN400409
*B. bigemina*	Kunming	*B. microplus*	JN400410
*B. bigemina*	Lushi	*B. microplus*	JN400411
*B. major*	Yili	*Haemaphysalis punctata*	JN400412
*B. ovata*	Lushi	*H. longicornis*	JN400413
*B. ovata*	Wenchuan	*H. longicornis*	JN400414
*B. ovata*	Zhangjiachuan	*H. longicornis*	JN400415
*Babesia* sp. Kashi2	Kashi	*Hyalomma spp.*	JN400416
*Theileria annulata*	Sanmenxia	*H. detritum*	JN400419
*T. annulata*	Xinjiang	*H. scupense*	JN400420
*T. annulata*	Ningxia	*H. detritum*	JN400428
*T. annulata*	Ankara	*H. detritum*	NC_011099
*T. sergenti*	Lushi	*H. longicornis*	JN400421
*T. orientalis*	Shintoku	*H. longicornis*	AP011947
*T. siensis*	Lintan	*H. qinghaiensis*	JN400422
*T. siensis*	Weiyuan	*H. qinghaiensis*	JN400423
*T. siensis*	Lintao	*H. qinghaiensis*	JN400427

### DNA extraction

The calves, aged between 12 and 24 months old, were infected by inoculating 5 ml of cryopreserved infected blood stock of these *Babesia* and *Theileria* isolates into the jugular vein. When the parasitemia reached 5 %, blood was collected into heparin vacutainer tubes via jugular venipuncture. The infected blood was resuspended in PSG buffer in the presence of SDS (final concentration was 2 %) and proteinase K (final concentration was 1 mg/ml). The solution was incubated at 42 °C for 14 h. Parasite DNA was extracted by conventional phenol/chloroform for deproteinization of the aqueous solution containing the desired nucleic acid. The purified DNAs were precipitated by the addition of two volumes of cold absolute ethanol. The pellet was dried, dissolved in sterile distilled water and kept at −20 °C until use. Control DNA was isolated from blood of uninfected cattle and blood of *Trypanosoma brucei evansi* infected mouse [19].

### PCR-RFLP analysis

To develop a PCR-RFLP technique for species- and strain-specific diagnosis of bovine *Babesia* and *Theileria* parasites, sequences conserved in all *Babesia* and *Theileria* species were identified from sequence alignment and used as primers in a single PCR protocol. For the PCR step, a PCR product that was about 707–855 bp long was amplified using primers 5′- ATGGGTATTTCACGTGACAG -3′ and 5′- GCGTTTCTTCTTATCCATACG -3′. Each PCR mixture (total volume, 50 μl) contained 5 μl of 10 × PCR buffer, 6 mM MgCl_2_, deoxynucleoside triphosphate at a concentration of 200 μM each, primer at a concentration of 200 nM each, 2.5 U of Taq polymerase, and 20 ng of DNA template. A total of 35 cycles, each consisting of 94 °C for 45 s, 54 °C for 45 s, and 72 °C for 1 min, were performed; an initial hot start at 94 °C for 3 min and a final extension step at 72 °C for 7 min were also included.

For restriction fragment analysis, 20 μl of the PCR products was digested in a 50-μl reaction mixture containing 20 U of M*bo*I (Takara) and 5 μl of the appropriate restriction buffer at 37 °C for 1 h, under conditions recommended by the supplier. The digested products were fractionated on a 3.0 % agarose gel and visualized by ethidium bromide staining. In additional, predicted restriction fragment length polymorphism (RFLP) patterns were produced in silico using the web-based software In Silico [20]. Image analysis of the electrophoretic gels was performed with 1-day Manager Software (TDI, Madrid, Spain).

## Results and discussion

### PCR-RFLP analysis

**Fig. 1:**
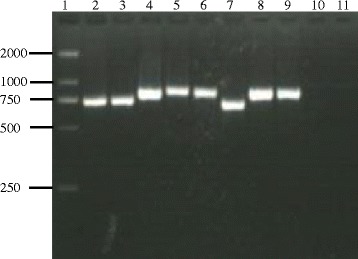
PCR products of a fragment of the RPS8 gene. Lane 1, 2000 bp size markers; lane 2: *T. sergenti*; lane 3, *T. annulata*; lane 4, *B. bovis*; lane 5, *B. major*; lane 6, *B. bigemina*; lane 7, *T. sinensis*; lane 8, *B. ovata*; lane 9, *Babesia* sp. Kashi 2; lane 10, *Trypanosoma brucei evansi*;lane 11, Negative control, cattle genomic DNA

**Fig. 2:**
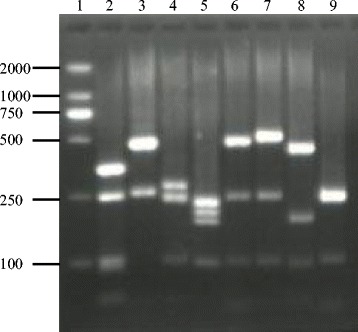
Fragments of the RPS8 (coding and non-coding regions) gene digested with *Mbo*I. Lane 1, 2000 bp size marker; lane 2, *B. bovis* (Shanxian and Lushi isolates); lane 3, *T. sergenti* (Lushi isolate); lane 4, *B. major* (Yili isolate); lane 5, *T. annulata* (Sanmenxia and Xinjiang and Ningxia isolates); lane 6, *Babesia* sp. Kashi (Kashi isolate) 2; lane 7, *B. bigemina* (Kunming and Lushi isolates); lane 8, *T. sinensis* (Weiyuan and Lintan and Lintao isolates); lane 9, *B. ovata* (Wenchuan and Lushi and Zhangjiachuan isolates)

PCR amplification of RPS8 gene from the DNA yielded a product of 709 bp for *T. annulata* isolates, 713 bp for *T. sergenti* Lushi isolate, 707 bp for *T. sinensis* isolates. Similarly, *Babesia* species yielded products that were similar or identical in size. PCR products of *B. bigemina* isolates, *B. major*, *B. ovata* isolates, *B. bovis* isolates, and *Babesia* sp. Kashi2 were 849, 847, 849, 820, 855 bp, respectively (Table 2, Fig. 1). Specificity for *Babesia* and *Theileria* was confirmed by the absence of products from samples of *Trypanosoma brucei evansi* and cattle genomic DNA (Fig. 1). The single PCR was quite sensitive (0.1 pg genomic DNA of *Babesia* species and 1 pg genomic DNA of *Theileria* species), as demonstrated by the amplification of serial diluted DNA samples (data not shown). Amplicon size alone could not distinguish the species. However, on digestion with M*bo* I, fragment polymorphism was visible post gel electrophoresis of the digested DNA (Table 2, Fig. 2). Thus RFLP will clearly distinguish among *Babesia-* and *Theileria-* infected cattles. However, this is based on a limited sample size and we need to confirm that there is no intra-specific restriction polymorphism, particularly for the complex *Theileria buffeli/orientalis* group [21, 22].

**Table 2:** The amplicon size, intron size, and Mbo I restriction fragment of RPS8 (coding and non-coding regions) genes of *Babesia* and *Theileria* species used in this study

Species	Strain	Amplicon size (bp)	Mbo I
*Theileria sergenti*	Lushi	713	464, 249
*T. orientalis*	Shintoku	713	464, 249
*T. annulata*	Xingjiang, Ningxia and Sanmenxia	709	227, 203, 182, 97
*T. annulata*	Ankara	709	227, 203, 182, 97
*T. sinensis*	Weiyuan, Lintao and Lintan	707	430, 182, 95
*Babesia bigemina*	Kunming and Lushi	849	506, 243, 100
*B. bovis*	Shanxian and Lushi	820	341, 243, 99, 90, 37
*B. major*	Yili	847	274, 243, 231, 99
*Babesia* sp. Kashi2	Kashi	855	476, 274, 99, 37
*B. ovata*	Zhangjiachuan, Wenchuan and Lushi	849	275, 242, 232, 99

A more practical assay is required to classify piroplasms such as *Theileria* and *Babesia* isolates since current serological and morphological tests cannot discriminate between closely related species [12]. Although reverse line blot (RLB) assay for the simultaneous identification of bovine *Babesia* and *Theileria* species has been developed, its use for routine diagnosis is restricted by various factors. These include the availability of reagents, complexity of operating procedures, special equipment needs and high susceptibility in the subjective interpretation of the hybridisation signal [23, 24]. Although nucleic acid-based tests such as real-time PCR and Loop-mediated isothermal amplification (LAMP) demonstrate significant sensitivity and specificity, they are only suitable for single species differentiation [25–29]. It would be desirable to have a ‘universal’ PCR-based test for the simultaneous detection and identification of these parasites. This requires the analysis of a molecular target conserved among piroplasms, yet variable enough to design a reliable species identification protocol.

In our previous study, RPS8 rDNA was confirmed to be a useful and novel genetic marker for defining species boundaries and for detecting closely related species, similar to 18S rDNA, because it tends to have little intra-species variation but considerable inter-species difference. It is relatively simple to amplify RPS8 rDNA by polymerase chain reaction (PCR) based on the highly conserved rDNA flanking both RPS8 regions [30, 31]. In this study, our results indicated that the RPS8-based PCR-restriction fragment length polymorphism was a simple and reliable molecular diagnostic tool for the simultaneous detection and identification of bovine *Babesia* and *Theileria* species in China, which could be applicable for the survey of parasite dynamics, epidemiological studies as well as prevention and control of the disease.

## Conclusions

In this work, we found that utilizing PCR with restriction fragment length polymorphism (RFLP) on the RPS8 gene can be useful for the differentiation of the most common pathogenic *Babesia* and *Theileria* species infecting cattles in China. However, more samples are needed to verify the usefulness of the RPS8 (coding and non-coding regions) gene as a marker for the detection of the most *Babesia* and *Theileria* species, particularly for some closely related species.
